# Genome-wide identification of the auxin/indole-3-acetic acid (Aux/IAA) gene family in pepper, its characterisation, and comprehensive expression profiling under environmental and phytohormones stress

**DOI:** 10.1038/s41598-018-30468-9

**Published:** 2018-08-13

**Authors:** Muhammad Waseem, Fiaz Ahmad, Sidra Habib, Zhengguo Li

**Affiliations:** 1 School of Life Sciences, Chongqing University, Shapingba, Chongqing China; 2 Institute of Pure and Applied Biology, Bahauddin Zakariya University, Multan, 60800 Pakistan

## Abstract

Auxin is an essential phytohormone that plays a crucial role in the growth and development of plants in stressful environments. Here, we analysed the auxin/indole-3-acetic acid (Aux/IAA) gene family, which produces auxin in pepper, and succeeded in identifying 27 putative members containing four conserved domains (I. II. III and IV) in their protein sequences. Sequence analysis, chromosomal mapping and motif prediction of all identified CaAux/IAA genes were performed. It was observed that these genes contained four conserved motifs divided into nine different groups and distributed across nine chromosomes in pepper plants. RNA-seq analysis revealed the organ specific expression of many CaAux/IAA genes. However, the majority of genes were expressed with high expression levels in the early stages of fruit development. However, the maximum expression level of the CA03g34540 gene was observed in the breaker stage. Moreover, thirteen CaAux/IAA genes were labelled as early responsive genes to various phytohormone and abiotic stresses. Furthermore, RNA-seq analysis in response to pathogen inoculation (PepMoV, TMV strains P0/P1, and *Phytophthora capsici*) showed distinct expression profiles of all identified genes, suggesting the diverse expression nature of genes under these stress conditions. Overall, this study provides insight into the dynamic response of CaAux/IAA genes under environmental and phytohormones stress conditions, providing bases to further explore the importance of these genes through mutant/transgenic analysis in pepper.

## Introduction

Environmental stresses (both biotic and abiotic) affect both crop production and quality. To cope with environmental hazards, plants have evolved several strategies, including the regulation of cellular processes by controlling gene expression through transcription factors (TFs)^1^. TFs can play vital roles in the regulation of concerted and complex physio-biological processes like growth and development through involvement in signalling pathways activated by various external and internal stimuli^2^. TFs are members of many gene families and are present in both plants and animals. In the plant genome, more than 60 different TF families have been studied^3^.

Auxin is a ubiquitous hormone that plays a role in many plant regulatory processes, including growth and development at the cellular, sub-cellular and whole plant levels. These proteins also play a pivotal role in plant responses to light, gravity, and temperature^4^. Auxin treatment can regulate rapid and precise gene expression. These genes belong to several auxin responsive classes, including transcription activators (ARF, auxin response factors), repressors (Aux/IAA, auxin/indole-3-acetic acid) and receptors (F-box proteins)^5^. Aux/IAA genes act as transcriptional repressors of ARF genes to regulate downstream auxin-regulated genes^6^. Moreover, these genes mediate multi phytohormone signalling pathways, such as the jasmonic acid^7^, salicylic acid^8^, ethylene^9^ and brassinosteroid pathways^10^.

The Aux/IAA proteins are nuclear localised^11^ with molecular weights ranging from 18 to 36 kD^12^. They are characterised by four highly conserved domains (I, II, III, IV) with 7–40 amino acid residues^13^. Aux/IAA proteins with four different domains are known as the canonical Aux/IAAs, while those lacking any of the four domains are known as the non-canonical Aux/IAAs. Domain I contains leucine-rich repeats (LxLxLx) and acts as a transcriptional repressor^10,14^. Domain II is responsible for Aux/IAA stability and interacting with F-box proteins (TIR1/AFB)^14^. Domains III and IV are involved in homodimerisation and heterodimerisation among Aux/IAAs and ARFs^15^.

To date, Aux/IAA genes have been explored and identified in a number of monocot and dicot plant species including *Carica papaya*^16^, *Cicer arietinum*^5^, *Eucalyptus grandis*^17^, *Solanum lycopersicum*^18,19^, *Solanum tuberosum*^20^, *Sorghum bicolor*^21^, *Arabidopsis thaliana*^22^, *Cucumis sativus*^23^, *Oryza sativa*^24^, and *Zea mays*^25^. Previously, most Aux/IAA genes were identified and characterised through mutant analysis in different crops. However, *Capsicum annum* L. has not been studied as much. In Arabidopsis, the auxin-insensitive mutant *iaa1/axr5* showed auxin-mediated growth effects^26^. Similarly, *iaa3/shy2* caused a significant effect on lateral root growth and formation, cell wall formation and homeostasis^27^. In tomato plants, the silencing of SlIAA9 and SlIAA17 produced pleiotropic developmental phenotypes^28^ and affected fruit size via endoreduplication in the pericarp^29,30^. In rice, *OsIAA9* or *OsIAA13* affected starch accumulation and were involved in lateral root intonation^31^.

Pepper is a member of night-shade family Solanaceae. It is very popular due to its pungency and its role as a rich source of vitamin C, and it is cultivated worldwide. The productivity of pepper is severely affected by a variety of pathogens, among them, soil borne diseases like *Phytophthora* blight, caused by *Phytophthora capsica*^4,32^. Several studies have revealed that resistance to this pathogen is polygenic, perhaps controlled through QTLs^1,15^. Many attempts have been made to identify and characterise candidate resistance genes and confer resistance against the pathogen. However, little is known about *capsicum-P*. *capsica* at the genetic and molecular levels^4^. This work revealed a comprehensive analysis of the Aux/IAA gene family in pepper plants grown under biotic and abiotic hazards. Using qRT-PCR and RNA seq, we created an organ specific expression profiles of all genes. Furthermore, the temporal expression profiles under various stress conditions (salt and drought) and phytochrome [IAA (indole-3-acetic acid), ABA (abscisic acid), JA (jasmonic acid), and GA (gibberellic acid)] were also created. Similarly, the expression patterns of identified genes against pathogens were assessed using RNA seq data^1^. The results indicate the vital role of CaAux/IAA genes against abiotic stresses and to *Phytophthora capsica*, *Pepper mottle virus*, and *Tobacco mosaic virus*. Our findings provide a foundation to explore more about these genes in pepper to better understand and explore the real role under different living and non-living environmental stresses.

## Results

### Pepper Aux/IAA gene sequence identification

To identify all Aux/IAA family members in the pepper genome, 29 AtIAAs and 26 SlAAs were queried in the SOL genome database and pepper genome database. Twenty-nine candidate genes were identified in the pepper genome. We also identified 27 candidate Aux/IAA genes in the pepper genome through the structural integrity of the conserved Aux/IAA domain (PF02309) in polypeptides by NCBI CDD and SMART. All verified genes were designated as Ca *(Capsicum annum)* Aux/IAA (CaAux/IAA) 1 to 27 in ascending order of their respective chromosome number. The peptide residue length, isoelectric point and relative molecular mass (in Daltons) varied greatly within the family, ranging from 100 aa/11.52 Da (CA01g18900) to 358 aa/38.36 Da CA04g17190 and from 5 pI (CA12g19830) to 10.31 pI (CA06g16770) (Table 1). Similar trends have been reported in Arabidopsis and tomato plants^18,22^. Such structural variability of CaAux/IAA may infer their functional diversity associated with various physio-biological processes during growth and development under a variety of environmental conditions. Similarly, the pairwise identity comparison of pepper Aux/IAA varies from as low as 7.69% (between CA04g00340 and CA07g13090) to a highly identical level between CA00g93260 and CA00g8288 of about 86.60% (Supplementary Fig. S1). The majority of putative CaAux/IAAs were localized in the nucleus, while a few were found in the cytoplasm and plastid (Table 1).

**Table 1:** The characteristics of the pepper Aux/IAA gene family.

Gene locus ID	Gene Name	Aa	Mr	pI	Grand average of hydropathicity (GRAVY)	Aliphatic index	Chromosome			Exon #	Sub-cellular Localization	Corresponding gene ID in Zunla-1
							Position	Start	End			
CA01g18900	Aux/IAA1	100	11.52	5.59	−0.249	65.3	1	157700917	157701337	2	Nucl	Capana01g001423
CA02g01930	Aux/IAA2	221	23.71	9.3	−0.505	72.4	2	16118700	16119529	3	Nucl	Capana02g000711
CA03g04310	Aux/IAA3	248	27.01	5.4	−0.497	66.85	3	10013927	10017352	5	Nucl	Capana03g004455
CA03g05890	Aux/IAA4	249	27.76	8.17	−0.338	81.81	3	15965231	15962175	4	Chlo	Capana03g004567
CA03g05910	Aux/IAA5	195	21.98	6.52	−0.783	66	3	16009467	16010743	3	Nucl	Capana03g004568
CA03g34530	Aux/IAA6	183	20.8	7.14	−0.577	68.63	3	253578857	253580858	2	Nucl	Capana03g000311
CA03g34540	Aux/IAA7	195	21.7	5.13	−0.514	74.92	3	253590720	25389704	4	Nucl	Capana03g000310
CA03g34660	Aux/IAA8	286	31.46	5.24	−0.465	71.22	3	253708165	253709895	4	Nucl	Capana03g000299
CA03g35880	Aux/IAA9	322	35.78	8.13	−0.5	74.47	3	255743681	255746592	4	Nucl	Capana03g000244
CA04g00340	Aux/IAA10	104	37.23	7.46	0.469	86.25	4	263022	264314	2	Mito	Capana06g002018
CA04g17190	Aux/IAA11	358	38.36	6.66	−0.36	77.09	4	209217545	209220368	5	Cyto	Capana04g000808
CA06g01310	Aux/IAA12	180	20.42	7.34	0.572	77.39	6	2769403	2771639	3	Nucl	Capana07g000990
CA06g01320	Aux/IAA13	194	22.08	7.66	−0.596	80.26	6	2782502	2784445	3	Cyto	Capana06g003073
CA06g10630	Aux/IAA14	190	21.33	6.06	−0.701	64.68	6	171327866	171328893	3	Nucl	Capana00g000236
CA06g13860	Aux/IAA15	146	17.1	6.96	−0.386	90.07	6	200975995	200976733	2	Cyto	Capana06g001465
CA06g16770	Aux/IAA16	114	12.98	10.34	−0.936	66.58	6	212364673	212365020	1	Nucl	Capana06g001308
CA06g16790	Aux/IAA17	102	12.22	9.62	−0.388	83.92	6	212394523	212394831	1	Chlo	Capana04g002496
CA06g27740	Aux/IAA18	106	17.78	8.28	−0.458	71.94	6	235773943	235774631	3	Nucl	Capana06g000110
CA07g04080	Aux/IAA19	188	20.74	4.44	−0.511	79.79	7	27416361	27417912	5	Cyto	Capana07g000391
CA07g13090	Aux/IAA20	163	18.36	9.1	−0.322	69.39	7	203589801	203590403	2	Chlo	Capana03g001065
CA09g06930	Aux/IAA21	166	18.38	5.32	−0.381	73.31	9	57300380	57301787	3	Chlo	Capana09g001096
CA11g00460	Aux/IAA22	195	22.73	4.77	−0.7	71.95	11	932299	935840	4	Nucl	Capana00g002644
CA11g08610	Aux/IAA23	213	23.19	9.45	−0.497	71.88	11	70116242	70117069	3	Nucl	Capana00g000845
CA12g19830	Aux/IAA24	239	26.44	5	−0.566	69.25	12	229148656	229152865	4	Nucl	Capana00g002758
CA00g43090	Aux/IAA25	197	22.4	7.87	−0.767	68.83	0				Nucl	Capana03g003343
CA00g82880	Aux/IAA26	236	26.07	6.52	−0.585	59.87	0				Nucl	Capana09g000285
CA00g93260	Aux/IAA27	224	23.5	8.57	−0.607	64.38	0				Nucl	Capana08g001238

aa, amino acids; pI, isoelectric point; MW, molecular weight. Nucl; Nucleus, Mito; Mitochondria, Cyto; Cytoplasm, Chlo; Chloroplast.

### Chromosomal localisation, multiple sequence alignment and phylogeny

**Figure 1:**
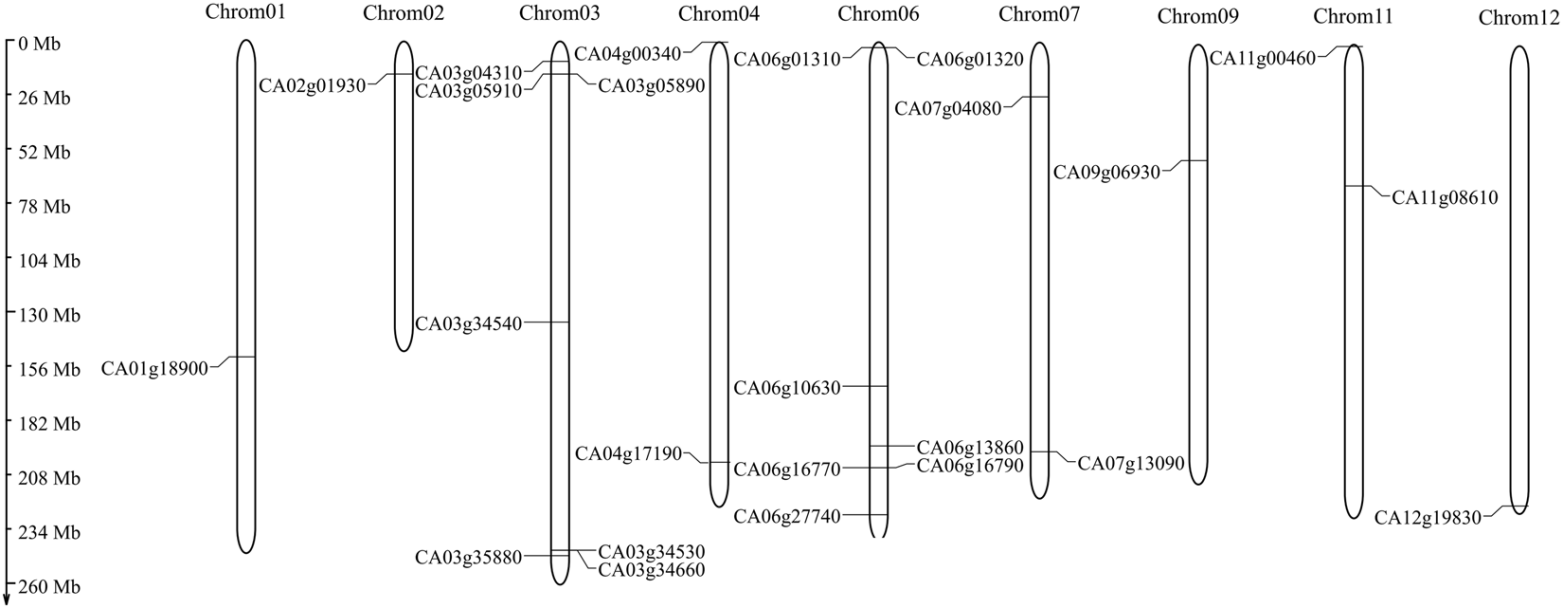
Chromosomal locations of pepper Aux/IAA genes. The locations of CaAux/IAA were based on physical locations. The numbers on the top indicate each chromosome number. The scale bar represents a 10 Mb chromosome distance.

**Figure 2:**
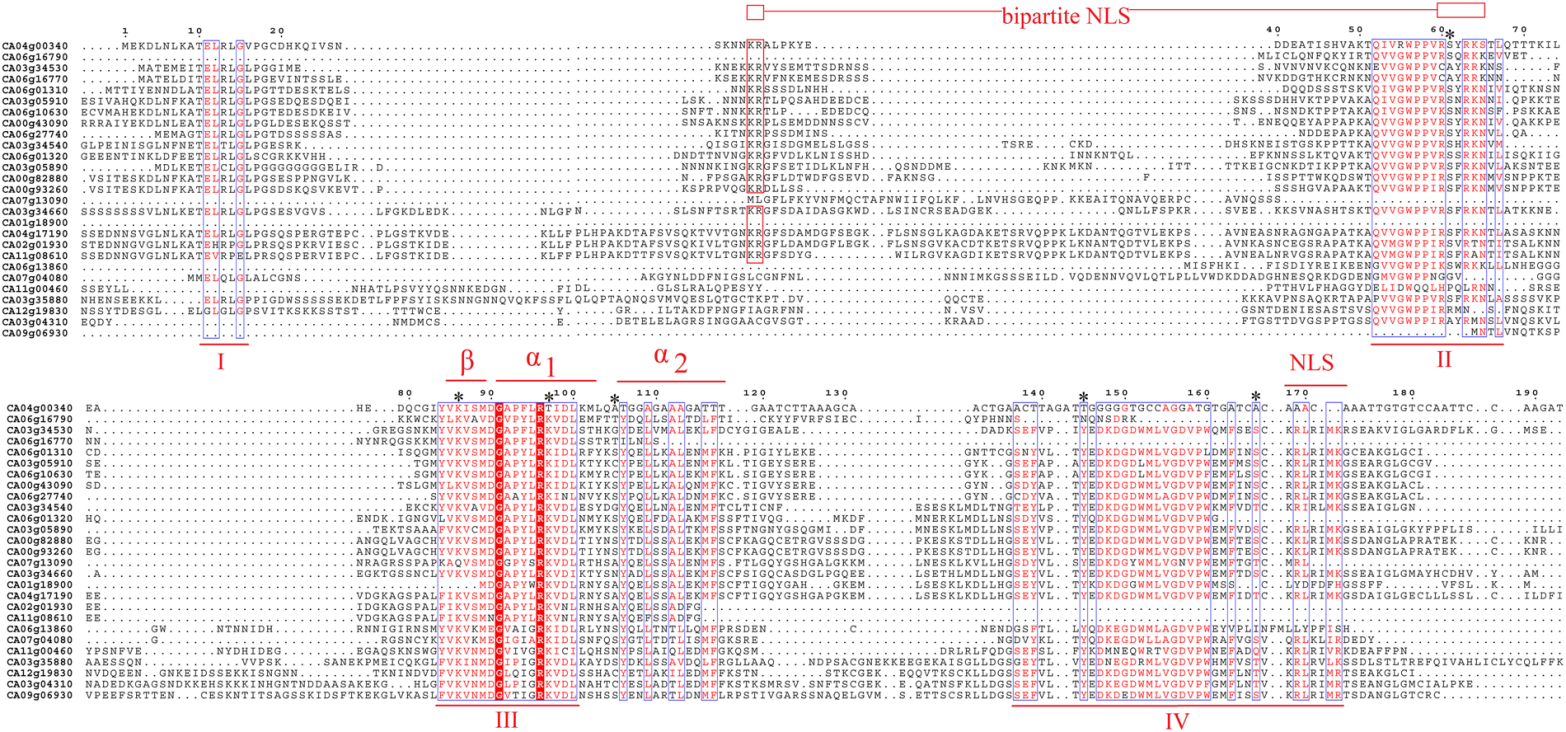
Multiple sequence alignment of 27 pepper Aux/IAA genes. Conserved domains I, II, III, and IV of the CaAux/IAAs are underlined. Nuclear localisation signals (NLSs) are indicated with red blocks. The βαα motif in domain III is marked with “β”, “α1”, and “α2”. The conserved amino acids are marked with asteroids*.

We performed *in silico* chromosome distribution of 23 CaAux/IAAs, excluding three genes (CA00g43090, CA00g82880, CA00g93260) located on unknown chromosomes, which were later assigned a position on any one of the 12 pepper chromosomes through refinements in pepper whole genome sequencing. All genes are located on different pepper chromosomes. However, no gene is located on chromosomes 5, 8 and 10. Seven CaAux/IAA genes are located on chromosome 3, while single genes are present separately on chromosomes 1, 2, 9 and 12 (Fig. 1). Multiple peptide sequence alignment revealed the existence of four highly conserved domains (I, II, III, IV) (Fig. 2) that were characteristic of canonical Aux/IAAs^33^; however, a few of them were on two conserved domains. The latter is known as non-canonical Aux/IAA and is short-lived^34^. In tomato, non-canonical Aux/IAA is expressed in certain development stages, suggesting their specific roles in auxin-mediated plant development^18^. Supplementary Table S1 showed the distribution of canonical and non-canonical Aux/IAA in different plant species. Domain IV was absent in CA02g01930, CA06g01310, CA06g16790, and CA11g08610, while domain I was absent in CA03g04310, CA06g16790, CA06g13860, and CA11g00460. Likewise, CA01g18900, CA07g13090, and CA09g06930 lack both domains I and II. Most CaAux/IAAs harbour two NLSs (Nuclear localisation signals). The bipartite NLS with one part was positioned between domains I and II, while another part near the terminus of domain II contained two stretches of R/K. Domain IV contained a second typical NLS that resembles SV40-type NLS. Moreover, βαα motifs that facilitate the dimerisation of Aux/IAA polypeptides were located in domain III. Kinase-specific phosphorylation sites were also predicted in CaAux/IAA proteins (Fig. 2).

**Figure 3:**
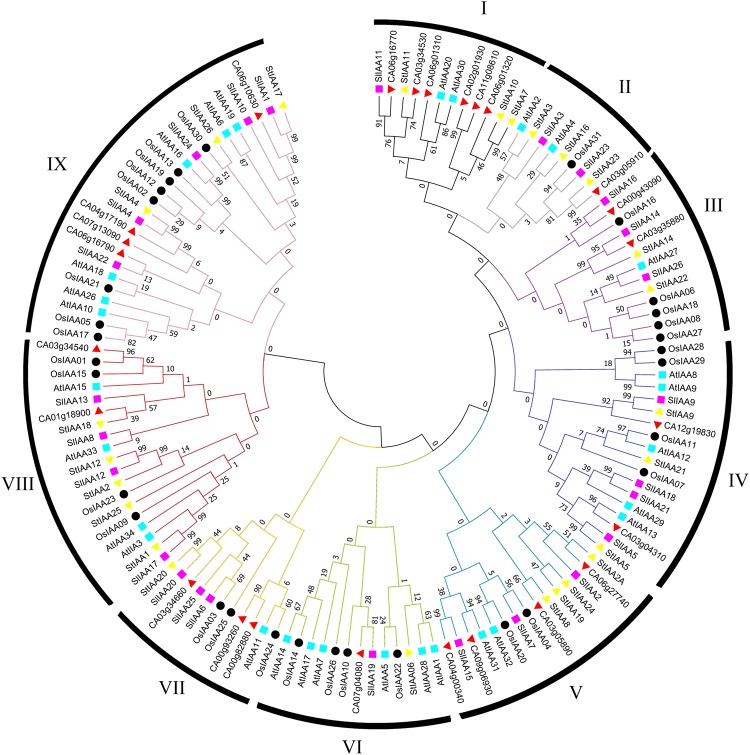
Phylogenetic relationship of pepper Aux/IAA proteins with Arabidopsis (*AtIAA*), rice (*OsIAA*), tomato (*SlIAA*), and potato (*StIAA*). Clustal Omega aligned the full-length amino acid sequences of Aux/IAA. The Neighbor-Joining phylogenetic tree with 1,000 bootstrap replicates was constructed using 137 Aux/IAA proteins. All Aux/IAAs are divided into nine groups, named I to XI.

In order to find the evolutionary relationship of pepper Aux/IAA with other plant species and to gain an understanding of the possible biological functions of this multi-gene family. A phylogenetic tree with 138 Aux/IAA peptide sequences from Arabidopsis, tomato, potato, rice, and pepper was constructed. These 138 Aux/IAAs were categorised into nine groups (I to IX). Group I contains six pepper Aux/IAAs. Groups II and VI contain one CaAux/IAA, while groups III, IV and VIII contain two CaAux/IAAs. Groups V and IX contain four pepper Aux/IAA genes each (Fig. 3).

### Analysis of motifs and phytohormone-related cis-regulatory elements

The C-terminal dimerisation domains (domains III and IV) of the IAA genes mediate homo- and heterodimerisation between and among IAAs and ARFs. Unlike tomato, a few pepper IAA genes lack one of these two C-terminal domains, including CA06g16770, CA06g16790, CA11g08610, and CA02g01930. The MEME was used to predict CaAux/IAA motifs in the peptide sequences. According to MEME analysis, the conserved domains of CaAux/IAA were divided into five conserved motifs; these were motifs 1 to 5, which have been predicted in most Aux/IAA proteins (Supplementary Fig. S2 and Table 2). Motif 1 is present in CA01g18900 and CA07g04080, while motif 5 is absent in CA03g04310. Similarly, CA02g01930, CA06g16770, and CA11g08610 contain motif 3 and motif 5, but these motifs are missing in CA09g06930. Meanwhile, motif 1 and motif 4 are missing in CA04g00340 and CA06g01320. CA06g13860 lacks motif 4 and motif 5, while motif 2 and motif 3 are present in CA06g16790. Motif 2 and motif 4 are present in CA11g00460, but CA07g13090 lacks motif 3, motif 4 and motif 5 (Supplementary Fig. S2).

**Table 2:** The identified consensus sequence of CaAux/IAA motifs by the MEME tool.

Motif name	Peptide residue length	Peptide sequence	Aux/IAA corresponding domain
Motif 1	29	LLNGSEYVLTYEDKDGDWMLVGDVPWEMF	Domain IV
Motif 2	41	GMYVKVSMDGAPYLRKVDLKMYKSYQELLSALEKMFSCFTG	Domain III
Motif 3	21	PTAKAQVVGWPPVRSYRKNTL	Domain II
Motif 4	20	INSCKRLRIMKGSEAKGLGC	Domain IV
Motif 5	19	LNLKATELRLGLPGSDSKE	Domain I

**Figure 4:**
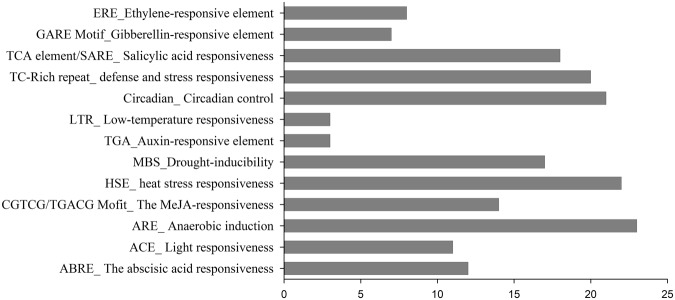
Total number of Cis-regulatory elements in the promoter sequences of the CaAux/IAA genes.

To explore the regulatory mechanism of pepper Aux/IAA genes to phytohormones, the cis-regulatory elements in the promoter region were analysed using online tools. A total of six hormone-related cis-regulatory elements were predicted, including abscisic acid (ABA), auxin (IAA), ethylene, salicylic acid (SA), gibberellic acid (GA) and methyl jasmonate (MeJA) (Supplementary Fig. S2 and Table S2). The presence of these putative cis-regulatory elements in pepper IAA implies their potential involvement in complex regulatory machines, including hormone signal transduction pathways. However, several other cis-regulatory sequences were also detected (Fig. 4 and Supplementary Fig. S4).

### Expression analysis of pepper Aux/IAAs in different organs

**Figure 5:**
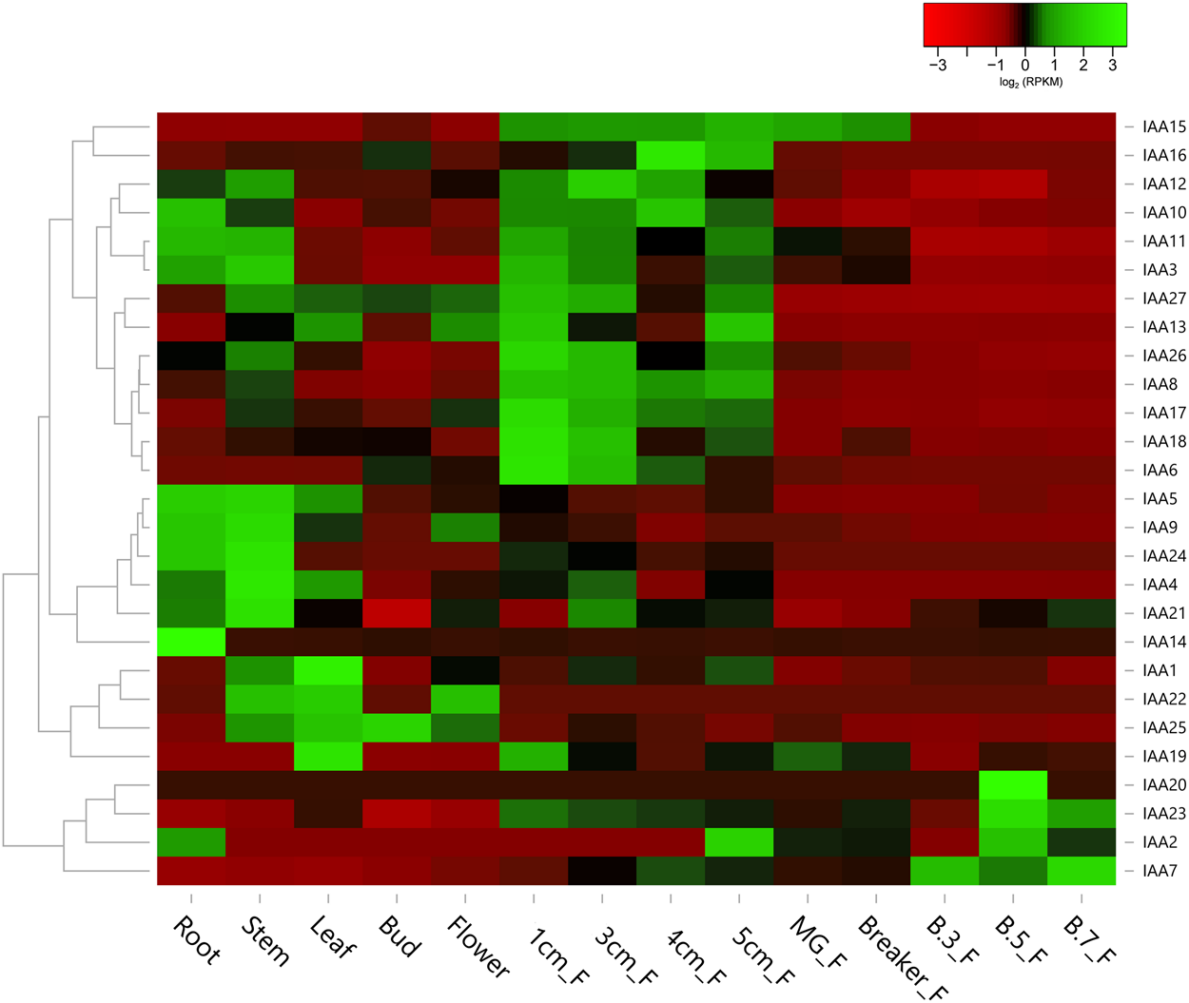
Organ specific expression analysis of CaAux/IAAs. The heat map was generated using RNA-seq from *C*. *annuum* ‘CM334’. Twenty-seven CaAux/IAA genes were used to construct the heat map. Red and green colours represent relatively low and high expression levels (log2 RPKM value), respectively. F, Fruit; MG_F, Mature green fruit; B.3_F, Breaker plus 3-day fruit; B.5_F, Breaker plus 5-day fruit, B.7_F, Breaker plus 7-day fruit.

We also analysed the differential expression patterns of all genes in various development stages using available RNA seq data^35,36^. The data included 14 plant organs, including root, stem, leaf, bud, and flower, as well as nine fruit development stages (Fig. 5). The pepper Aux/IAAs exhibited an organ specific expression profile (Fig. 5 and Supplementary Table S3) with most of them expressed at high levels in the fruit at different development stages. CA04g00340, CA04g17190, CA06g10630, and CA03g05910 were all specifically expressed in the root and contained root-specific cis-regulatory elements, suggesting their role in root development. CA03g04310, CA03g05890, CA03g05910, and CA03g35880 showed higher transcript accumulation in stems, suggesting their role in stem development. Similarly, higher transcript levels of CA01g18900 and CA07g04080 were found in leaf and CA00g43090 in the flower bud. Most pepper Aux/IAA genes were expressed in descending levels of expression from 1 cm fruit to B + 7-day fruit, while only a single gene (CA03g34540) was expressed in ascending order, with its expression having the highest expression at B + 7 fruit (RPKM = 1616.39) (Supplementary Table S3). To validate the RNA-seq results, we performed organ specific expression profiling of all pepper Aux/IAAs using qRT-PCR (Supplementary Fig. S5). The expression profile obtained through qRT-PCR was in consistent with the RNA-seq data.

### Expression analysis in response to abiotic stresses

**Figure 6:**
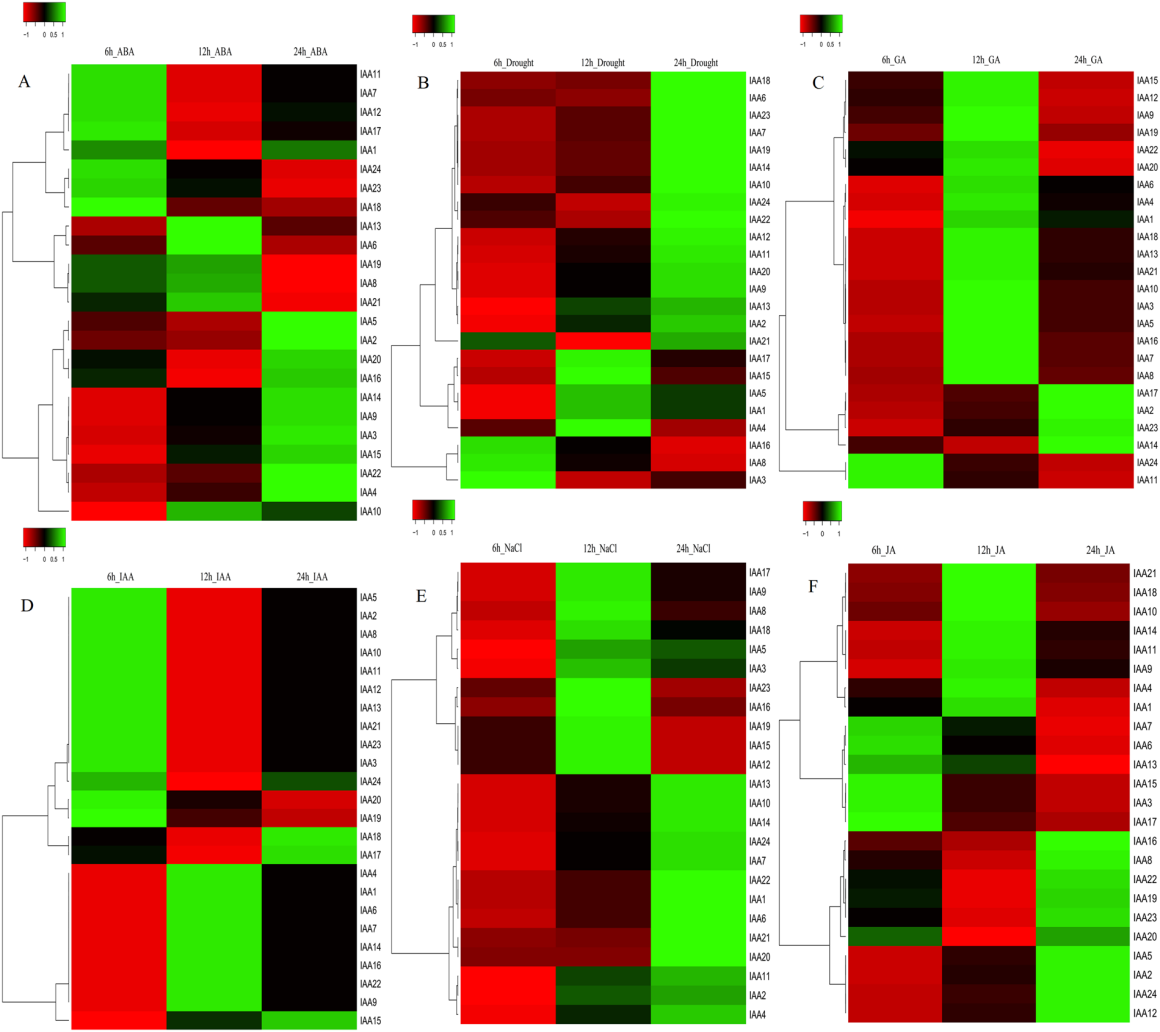
Temporal differential expression of CaAux/IAAs under abiotic and phytohormone stresses. Twenty-seven CaAux/IAA genes were used to construct each heat map. (**A**) ABA; (abscisic acid), (**B**) Drought, (**C**) GA; (gibberellic acid), (**D**) IAA; (Inode-3-acetic acid), (**E**) NaCl, and (**F**) JA; (jasmonic acid) at 6 h, 12 h and 24 h. Red and green colours represent relatively low and high expression levels (log2 RPKM value), respectively.

Many studies have shown that pepper plants are very sensitive to abiotic stresses, including those related to drought, salinity, and temperature. We thus investigated the potential role of CaAux/IAAs under salt and drought stress conditions. The pepper Aux/IAA genes were insensitive to salt treatment at 3 h but showed enhanced expression levels under conditions of prolonged treatment. Following drought treatment, CaAux/IAAs showed temporal expression levels. CA03g04310, CA03g34660, and CA06g16770 were induced only at 6 h, while CA01g18900, CA03g05890, CA03g05910, CA06g13860, and CA06g16790 were all highly expressed after 12 h under drought conditions (Fig. 6B,E).

### Expression profiling of the CaAux/IAA gene family in response to phytohormones

Auxin was the first plant hormone identified that regulates various aspects of plant growth and development. We analyzed the expression patterns of Aux/IAA genes to exogenous auxin. To this end, pepper seedlings were exposed at different time sets during treatment. The results revealed the temporal expression of the CaAux/IAA gene family. The transcript levels of CA06g13860, CA06g16790, and CA06g27740 increased temporally from 6 h to 24 h; however, CA07g04080 and CA07g13090 showed antagonistic expression patterns. These results indicated that a majority of CaAux/IAA genes responded to IAA treatment (6 h) and could be considered to be auxin-sensitive genes (Fig. 6D). To explore whether the CaAux/IAA genes are involved in multiple hormone signalling pathways, we performed and analysed expression profile analyses of these genes under various hormone treatment conditions including ABA (abscisic acid), JA (jasmonic acid), and GA (gibberellic acid). Following the ABA treatment, several genes were up and down-regulated. CA03g34540, CA04g00340, CA04g17190, CA06g01310, CA06g01320, and CA06g16790 were down-regulated temporally, while CA02g01930, CA03g04310, CA03g05890, CA03g05910, CA06g10630, CA06g13860, CA06g16770, CA07g13090, and CA11g00460 were up-regulated after 24 h (Fig. 6A). CA04g17190 and CA12g19830 showed enhanced expression at 3 h after GA treatment (Fig. 6C); however, CA02g01930, CA06g10630, CA06g16790, and CA11g08610 showed late expression at 24 h after GA treatment. JA induced temporal down-regulation (CA03g04310, CA03g34530, CA03g34540, CA06g01320, CA06g13860, CA06g16790) and up-regulation (CA02g01930, CA03g05910, CA03g34660, CA06g01310, CA06g16770, CA07g13090, CA11g00460, CA11g08610, CA12g19830) of the CaAux/IAA genes (Fig. 6F). Furthermore, CA02g01930 was the only gene that was expressed temporally (low at 6 h, high at 24 h) in all hormone treatments (ABA, JA, GA). These results indicated that the expression levels of many CaAux/IAA genes were affected in an antagonistic manner in response to different hormones. Genes expressed 6 h after treatments are regarded as early response (ER) genes, while those expressed 24 h after treatments are instead regarded as late response (LR) genes. A gene under one treatment acts as an ER and as an LR under another treatment. For example, CA12g19830 was sensitive to ABA and GA treatments (at 6 h), while acting as a LR gene under JA stress. Similarly, CA06g01310 and CA11g08610 responded as ER genes for ABA but instead acted as an LR under JA. CA06g13860, CA03g04310 acted as an ER and LR under JA and ABA, respectively; however, CA06g16790 was induced as an ER (Fig. 6A,C,F). These results revealed that pepper Aux/IAA genes are possibly involved in multiple phytohormone signalling pathways.

### The response of CaAux/IAAs to pathogen inoculation

**Figure 7:**
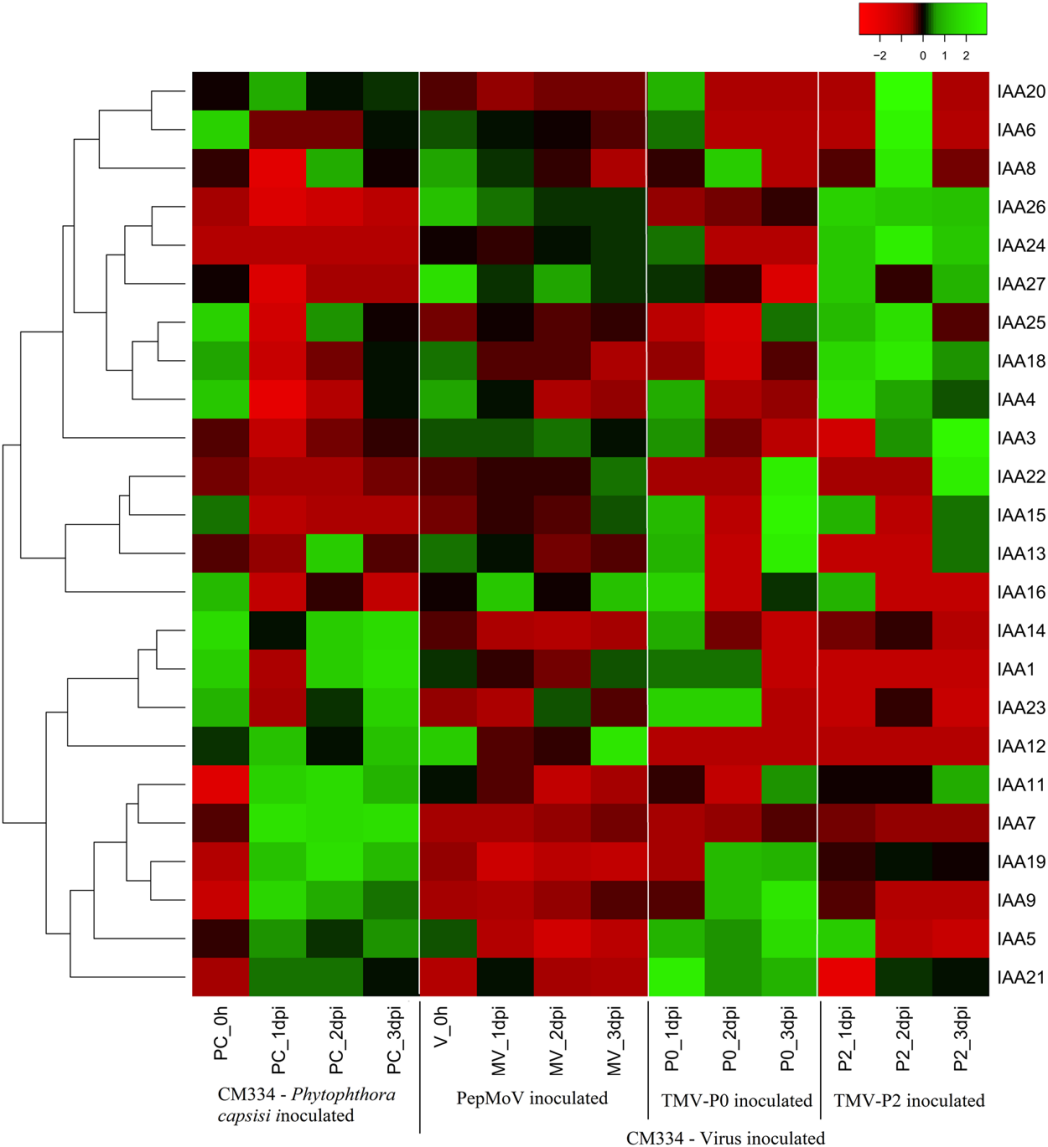
Differential expression analysis of CaAux/IAAs challenged under biotic stress. The heat map was generated using RNA-seq from three viruses (TMV-P0, TMV-P2, and PepMoV) and *Phytophthora capsica* inoculated *C*. *annuum* ‘CM334’. The 24CaAux/IAA genes were used to construct the heat map. The 3 CaAux/IAAs, which are absent data, were excluded. The data were normalised using control samples [Mock_virus (V_0h) and Mock_P.c (PC_0h)]. Red and green colours represent relatively low and high expression levels (log2 RPKM value), respectively. P.c, *Phytophthora capsici*; D, days post inoculation.

To investigate the differential expression patterns of CaAux/IAA genes in susceptible and resistant responses against TMV-P2 and TMV-P0, respectively, we retrieved and generated a heat map based on publicly available RNA-seq data of pepper^1^ plants inoculated with TMV pathotype 0 (TMV-P0), TMV-P2, PepMoV, and *P*. *capsica*. The expression profile of each CaAux/IAA exhibited a unique expression pattern in response to inoculation (Fig. 7). In response to *P*. *capsica*, 13 CaAux/IAA genes were up-regulated, including CA04g17190, which showed high expression levels compared to the mock and other virus inoculations. Two genes, CA03g34540 and CA07g04080, showed particularly strong expression levels against *P*. *capsici* inoculation. CA03g05890 and CA04g17190 were highly up-regulated in the susceptible and resistant responses against TMV-P2 and TMV-P0, respectively. In contrast, the expression levels of the two CaAux/IAAs were repressed in P1 CA00g93260 and CA01g18900. However, some CaAux/IAA genes (CA03g05890, CA03g05910, CA04g17190, CA07g04080, CA00g43090) were up-regulated against both responses. After PepMoV inoculation, some pepper IAA genes were down-regulated, including CA03g05890, CA03g05910, CA04g17190, and CA07g04080. CA04g17190 showed decreased expression levels after inoculation with PepMoV but enhanced expression after inoculation with other pathogens. On the other hand, CA00g93260 showed down-regulation after inoculation with all pathogens (Fig. 7, Supplementary Table S4).

## Discussion

Pepper is an important vegetable crop of the Solanaceae family, along with tomatoes and potatoes. It is famous for its pungency and is a major ingredient of cuisines across the globe. Auxin is an essential phytohormone that regulates a number of plant growth and development processes. In pepper plants, no systematic knowledge of the auxin-related gene Aux/IAA has been reported. However, genome-wide studies of the Aux/IAA gene family have been done in other several monocot and dicot plant species, including *Carica papaya*^16^, *Cicer arietinum*^5^, *Eucalyptus grandis*^17^, *Solanum lycopersicum*^18,19^, *Solanum tuberosum*^20^, *Sorghum bicolor*^21^, *Arabidopsis thaliana*^22^, *Cucumis sativus*^23^, *Oryza sativa*^24^, *Zea may s*^25^, *Populustrichocarpa*^37^, *Brassica rapa*^38^, *Glycine max*^5^, and *Brassica napus*^39^. The number of genes in plants varies from 18 in *Carica papaya*^16^ to 117 in *Brassica napus*^39^ (Supplementary Table S1). Furthermore, many polypeptides have been reported to be involved in plant morphogenesis, fruit development, and tropic responses. In this study, we reported a brief organ specific expression profile and the first systematic investigation of biotic responses to the Aux/IAA gene family in peppers. A total of 27 CaAux/IAA genes were identified in the pepper genome that are very similar to those reported in the Arabidopsis^22^, tomato^18,19^ and rice^24^ genomes.

Through the phylogenetic analysis of 27 peppers, Aux/IAA genes with 110 peptides from tomato, Arabidopsis, potato and rice using the NJ method were divided into nine groups, with each group including one to five pepper Aux/IAA genes. Group-I contained five genes, while group II and VI had one CaAux/IAA gene each (Fig. 2). Gene structure analysis revealed that CaAux/IAA contained very few introns, ranging from 1 to 5 (Supplementary Fig. S6). Similar trends have been reported in Arabidopsis^22^, tomato^18,19,40^, potato^20^, and rice^24^ species, suggesting their evolutionary conservation. *In silico* analysis of the chromosome location of CaAux/IAA showed that all genes are localised on 9 of the 12 pepper chromosomes. Chromosome 3 and 6 contained seven genes on each, while chromosomes 1, 2, 9, and 12 each contained only one gene (Fig. 1).

The temporal regulation of organ specific transcript abundance is important in the transcriptional control of gene expression that leads to different growth, development, abiotic and biotic responses in plants. Over the past decade, RNA-seq has become a potent and effective tool for the analysis of gene expression. RNA seq analysis showed the organ specific expression of CaAux/IAA among selected samples. Most CaAux/IAA genes showed organ specific expression, while others had different expression patterns in various organs. CA03g34540, CA03g34530, CA06g16790, CA04g17190, CA00g93260, and CA03g34530 were expressed at high levels at various developmental stages. Nine and three out of twenty-seven CaAux/IAA genes showed high expression levels in 1 cm fruit and root. Similarly, 10 SlAux/IAA and 6 StAux/IAA genes were expressed at relatively high levels in root, while very few genes were expressed in 40-day-old tomato fruit^20,40^. The relative expression levels of thirteen CaAux/IAA genes was high in fruits at various developmental stages (Fig. 5, Supplementary Fig. S5, Supplementary Table S3). Furthermore, CA03g34540 showed high expression levels among all pepper Aux/IAA genes in seven-day-old breaker fruit, suggesting that this gene could play a role in pepper fruit development and ripening. Thus, differentially regulated CaAux/IAA genes may play a key role in pepper fruit growth, development, and ripening.

Aux/IAA genes are nuclear-localised, short-lived and induced in response to auxin in several plant species, including tomato, sorghum, and Arabidopsis, where they are expressed differentially^14,41^. Several CaAuX/IAA genes are sensitive to exogenous auxin, while others remain insensitive to prolonged stress. Additionally, CA04g17190, CA03g34540, CA06g01310, CA06g01320, CA06g13860, CA06g16790, CA11g08610, and CA12g19830 were able to respond quickly to other phytohormones, including ABA (abscisic acid), GA (gibberellic acid) and JA (jasmonic acid) (Fig. 6A,C–E). However, research has shown that several plants showed auxin-mediated responses to various abiotic factors^42,43^ in maize, Arabidopsis and rice^25,44,45^. Here, we performed temporal expression profiling of CaAux/IAA under drought and salt (NaCl) stress. CA03g04310, CA03g34660, and CA06g16770 were sensitive to water deficiency. However, the majority of genes were observed to act as late responsive genes to salt and drought stresses (Fig. 6B,E).

Not much has been conclusively reported about Aux/IAA genes, especially under biotic stress conditions. Our study unveiled the distinct temporal expression pattern against pathogen inoculation. It was predicted that the gene CA03g05890 showed changes in expression against the susceptible response to TMV-P0, while CA09g17190 showed a resistant response against *P*. *capsica* and TMV-P0, and decreased expression to PepMoV. However, CA00g329260 was down-regulated in response to all pathogens. Differentially expressed CaAux/IAAs might be involved in the defence responses against biotic stress (Fig. 7). Our study bridges the knowledge of Aux/IAA genes in Solanaceae and helps to improve our understanding about the possible involvement of these genes in plant growth and development under various phytohormone and biotic stresses.

## Methods

### Identification and characterisation of the Aux/IAA gene family in pepper plants

To retrieve pepper Aux/IAA peptide sequences, 29 Arabidopsis and 26 tomato protein sequences were obtained from the TAIR genome database and Sol genomic database, respectively. These were used as query sequences in the Pepper Genome (http://peppergenome.snu.ac.kr/), PGD (Pepper Genome Database, http://peppersequence.genomics.cn/page/species/index.jsp) and SOL genomic database for pepper plants (https://solgenomics.net/organism/Capsicum annuum/genome) for homology searches^35,36^. All putative sequences were subjected to domain searches in the NCBI CDD (conserved domain database, https://www.ncbi.nlm.nih.gov/Structure/cdd/wrpsb.cgi)^46^ and SMART database (http://smart.emblheidelberg.de/)^47^. Identified pepper Aux/IAA genes were further subjected to Compute pI/Mw (https://web.expasy.org/compute_pi/)^48^ to determine the peptides’ molecular weight and pIs (isoelectric points). Meanwhile, *in-silico* putative sub-cellular location analysis was performed using the WoLFPsort server (https://wolfpsort.hgc.jp/)^49^.

### Multiple sequence analysis (MSA) and phylogeny

The ClustalO (https://www.ebi.ac.uk/Tools/msa/clustalo/) program was used to perform an MSA of putative pepper Aux/IAA sequences using default parameters^50^. Phylogenetic analysis was performed using 137 Aux/IAA peptides (27 Arabidopsis, 26 tomato, 31 rice and 26 potato) including 27 pepper Aux/IAAs in MEGA 7.0^51^ by an NJ (neighbour joining) method. The bootstrap was set at 1000 replicates.

### Chromosome mapping, gene structure, cis-regulatory elements and motif analysis

The chromosome position of each Aux/IAA gene was mapped through the SOL genomic database using the MG2C v.2 program (http://mg2c.iask.in/mg2c_v2.0/). The retrieved genome and CDS sequences from the SOL genome database were put into GSDS 2 (Gene Structure Display Server, http://gsds.cbi.pku.edu.cn/^52^ to display exon-intron distribution in the gene. The plant CARE database (http://bioinformatics.psb.ugent.be/webtools/plantcare/html/) for cis-regulatory elements were used to identify possible putative hormone-related cis-regulatory elements present in the promoter region of genes^53^. The new PLACE database (https://sogo.dna.affrc.go.jp/cgi-bin/sogo.cgi?sid=&lang=ja&pj=640&action=page&page=newplace) was used to verify predicted regulatory motifs^54^. Approximately ~1000 nucleotide sequences from the 5′UTR (before ATG) were downloaded from the SOL genome for each gene. The conserved motifs were visualised using MEME V.4.12.0^55^ of pepper Aux/IAA proteins with parameters set at (i) zero or one occurrence of single motif per sequence, (ii) motif width up to 250, and (iii) maximum number of motifs per sequence set to 5, while other parameters set to default.

### Plant growth, stress treatment, and sample preparation

The sterilised seeds of pepper (*Capsicum annum* L.) were sown in a greenhouse under 16 h/8 h light/dark cycle with 26 °C/18 °C day/night temperatures and with a light intensity of 350 µmolm^−2^s^−1^. Plants were regularly irrigated with nutrient media. For organ specific profiling, plant parts like root, stem and leaves from six-week-old plants were selected. Flowers were collected after opening, and fruits were harvested at mature green stage^1^.

For stress and phytohormone treatment, plants with 6–8 true leaf stages were used. For salt treatment, 200 mM NaCl was used. For drought, unrooted fully hydrated seedlings were placed on filter pepper. For phytohormone treatments, leaves were sprayed with 10 µM IAA, 10 µM ABA, 10 µM GA, and 10 µM JA^2^. Plants treated with water were used as the controls. Leaves from plants were harvested at 0, 6, 12, and 24 hours and immediately stored at −80 °C until further analysis. All the experiments were repeated three times.

### RNA isolation, cDNA preparation, and qRT-PCR analysis

Tissue (0.1 g) was used for total RNA extraction using an E.Z.N.A.® Plant RNA Kit following the manufacturer’s protocol. The RNA was quantified and qualified by using a thermo-scientific NanoDrop. The first complementary strand of DNA was synthesised using a PrimeScript™ first strand cDNA Synthesis Kit according to the manufacturer’s protocol. A 96-well reaction mixture plate was used to perform qRT-PCR on a BIO-RAD CFX manager with gene primers. The reaction mixture contained 5 µl of 2 × SYBR® Premix Ex Taq™, 1 µl of forward and reverse primers each, 1 µl of cDNA and 2 µl of ddH_2_O. The reaction system was set as 95 °C pre-denaturation for 1 minute, 95 °C for 5 seconds, 39 cycles of primer annealing (according to primer Tm) for 30 seconds each, followed by 65 °C post-denaturation for 5 seconds. Three independent replicates were used. Pepper *actin* (CA12g08730) was used as the internal control. The relative expression level was calculated using the 2^−DDCt^ method, and a heat map was drawn using HemI 1.0^56^.

### Transcriptome analysis

To analyse the organ specific expression profile of CaAux/IAAs at various development stages, we obtained previously generated RNA-seq data of pepper plants^35,36^, including root, stem, leaf, bud, flower, and fruit. The fruit from nine development stages was selected, including five pre-breaker stages and three post breaker stages. Similarly, to analyse the expression pattern of CaAux/IAA in response to pathogen infection, we retrieved RNA-seq data from previous researchers^1^, including tobacco leaves infected with TMV-P0, TMV-P2, PepMoV, and *P*. *capsici*. For expression profiling, Reads Per Kilobases per Million mapped reads (RPKM) values from RNA-seq data were log^2^ transformed. Expression patterns with hierarchical clustering are displayed in Heatmapper (http://www1.heatmapper.ca/expression/).

## Electronic supplementary material

Supplementary information
