# Supplementary material for: Genome-wide identification of the auxin/indole-3-acetic acid (Aux/IAA) gene family in pepper, its characterisation, and comprehensive expression profiling under environmental and phytohormones stress
Source: Sci Rep. 2018 Aug 13;8:12008. doi: 10.1038/s41598-018-30468-9 (PMC6089902; doi:10.1038/s41598-018-30468-9)

**Genome-wide identification of the auxin/indole-3-acetic acid (Aux/IAA) gene family in pepper, its characterisation, and comprehensive expression profiling under environmental and phytohormones stress**

Muhammad Waseem^1^, Fiaz Ahmad^2^, Sidra Habib^1^, Zhengguo Li^1*^1

1School of Life Sciences, Chongqing University, Shapingba, Chongqing, China

2Institute of Pure and Applied Biology, Bahauddin Zakariya University, Multan, Pakistan

*^*^Corresponding author:*

*Zhengguo Li****^1*^***

*Email:* [*zhengguoli@cqu.edu.cn*](mailto:zhengguoli@cqu.edu.cn)*,* [*lizhengguo.cqu@outlook.com*](mailto:lizhengguo.cqu@outlook.com)

**Supplementary information**

**Supplementary Tables**

**Table S1.** Numbers of Aux/IAA family gene members in angiosperm species along with pepper. The genes were divided into two categories (canonical and non-canonical) based on the presence or absence of four Aux/IAA conserved domains (I, II, III, and IV).

**Table S2.** Putative cis-elements of CaAux/IAA promoters. The 1000 5`UTR from ATG was subjected to the Plant CARE and PALACE database for cis-regulatory elements prediction.

**Table S3.** RPKM values of pepper genes against RNA-seq data of pepper Aux/IAAs (NCBI accession No. GSE45037).

**Table S4.** RPKM values of pepper Aux/IAA genes against RNA-seq data of pathogen-infected tissues.

**Supplementary Figures**

**Figure S1.** Sequence identity matrix of pepper Aux/IAA proteins.

**Figure S2.** Motif distributions in the pepper Aux/IAA genes. The MEME web server was used to predict the motif distribution of the Aux/IAAs. Each coloured box represents a specific motif.

**Figure S3.** Cis-elements in the promoters of CaAux/IAA genes for phytohormone responses. The CaAux/IAAs phytohormone responsive cis-regulatory sequences include ABA (abscisic acid), MeJA (Methyl Jasmonic acid), IAA (Indole-3-acetic acid), SA (salicylic acid), GA (gibberellic acid) and ethylene are represented by the blue, red, yellow, green, black and pink downward triangles, respectively.

**Figure S4.** Cis-elements in the promoter regions of each CaAux/IAA gene.

**Figure S5.** The qRT-PCR analysis of the tissue-specific expression of CaAux/IAAs. Twenty-seven CaAux/IAA genes were used to construct the heat map. Red and green colours represent relatively low and high expression levels (log2 RPKM value), respectively.

**Figure S6.** Gene structure (Exon/intron) of pepper Aux/IAA genes. Yellow boxes represent exons, and black lines represent introns. The scale bar on the bottom line indicates the size of the genes.

| **Species** | **Number of Aux/IAA genes** | **canonical** | **non- canonical** | **Reference** |
| --- | --- | --- | --- | --- |
| *Capcicum annum* | 27 | 16 | 11 | This study |
| *Carica papaya* | 18 | 10 | 8 | Liu et al. 2017 |
| *Cicer arietinum* | 23 | 17 | 6 | Singh et al. 2015 |
| *Eucalyptus grandis* | 24 | 17 | 7 | Yu et al. 2014 |
| *Solanum lycopersicum* | 25 | 23 | 2 | Audran-Delalande et al. 2012 |
| *Solanum tuberosum* | 26 | 21 | 5 | Gao et al. 2016 |
| *Sorghum bicolor* | 26 | 24 | 2 | Wang et al. 2010 |
| *Arabidopsis thaliana* | 29 | 23 | 6 | Overvoorde et al. 2005 |
| *Cucumis sativus* | 29 | 25 | 4 | Gan et al. 2013 |
| *Oryza sativa* | 31 | 25 | 6 | Jain et al. 2006 |
| *Zea mays* | 34 | 13 | 18 | Ludwig et al. 2013 |
| *Populus trichocarpa* | 35 | 29 | 6 | Kalluri et al. 2007 |
| *Brassica rapa* | 55 | 44 | 11 | Paul et al. 2016 |
| *Glycine max* | 63 | 50 | 13 | Singh et al. 2015 |
| *Brassica napus* | 119 | 76 | 43 | Li et al. 2017 |

| ***Cis*-element** | **PLACE motif identity** | **Core motif** | **Gene ID** | **Biological function** |
| --- | --- | --- | --- | --- |
| -300CORE | S000001 | TGTAAAG | CA03g34540 | Seed, endosperm storage protein |
| 2SSEEDPROTBANAPA | S000143 | CAAACAC | CA06g01310, CA06g01320 | Storage protein, seed |
| AACACOREOSGLUB1 | S000353 | AACAAAC | CA03g05910, CA03g34530, CA04g00340, CA07g13090 | endosperm-specific expression |
| ARFAT | S000270 | TGTCTC | CA07g04080, CA01g18900, CA04g00340, CA06g16790, CA06g27740, CA11g00460, CA02g01930 | Auxin response element (AuxRE1) |
| ARR1AT | S000462 | CTCTT | CA03g05890, CA03g04310, CA03g05910, CA06g01320, CA06g10630, CA09g06930, CA01g18900, CA03g34530, CA03g34660, CA03g35880, CA04g00340, CA04g17190, CA06g01310, CA06g13860, CA06g16770, CA06g16790, CA06g27740, CA07g13090, CA11g00460, CA11g08610, CA12g19830, CA02g01930 | Nodule |
| CATATGGMSAUR | S000370 | CATATG | CA06g01320, CA07g04080, CA09g06930, CA03g34530, CA03g35880, CA04g00340, CA04g17190, CA06g13860, CA06g27740, CA11g00460, CA12g19830, CA02g01930, | Auxin-responsiveness |
| DRE2COREZMRAB17 | S000402 | ACCGAC | CA03g34530, CA06g13860 | Drought |
| GARE1OSREP1 | S000419 | TAACAGA | CA07g04080, CA04g00340, CA06g27740 | Gibberellin-responive, seed |
| GCN4OSGLUB1 | S000277 | TGAGTCA | CA03g35880 | Endosperm |
| GT1GMSCAM4 | S000453 | GAAAAA | CA03g05890, CA03g04310, CA06g01320, CA06g10630, CA07g04080, CA09g06930, CA01g18900, CA03g34530, CA03g34660, CA03g35880, CA06g13860, CA06g27740, CA07g13090, CA12g19830 | salt-induced gene expression |
| LEAFYATAG | S000432 | CCAATGT | CA01g18900 | Root apical meristem |
| LTRE1HVBLT49 | S000250 | CCGAAA | CA06g13860, CA07g13090 | low-temperature-responsive elemen, cold |
| LTREATLTI78 | S000157 | ACCGACA | CA06g13860 | low-temperature-responsive elemen, cold |
| LTRECOREATCOR15 | S000153 | CCGAC | CA03g05910, CA06g01320, CA03g34530, CA03g34660, CA04g00340, CA06g13860 | low-temperature-responsive elemen, cold |
| MYCATERD1 | S000413 | CATGTG | CA09g06930, CA03g34530, CA04g00340, CA11g00460 | Dehydration, water stess |
| MYCATRD22 | S000174 | CACATG | CA03g05890, CA03g04310, CA01g18900, CA03g34530, CA03g35880, CA04g00340, CA06g01310, CA11g00460 | Dehydaration, water stess |
| NODCON1GM | S000461 | AAAGAT | CA03g05890, CA03g04310, CA03g05910, CA06g01320, CA09g06930, CA03g34530, CA06g16790, CA11g00460, CA11g08610, CA02g01930 | Nodule |
| OSE1ROOTNODULE | S000467 | AAAGAT | CA03g05890, CA03g04310, CA03g05910, CA06g01320, CA09g06930, CA03g34530, CA06g16790, CA11g00460, CA11g08610, CA02g01930 | Root, nodule, arbuscule |
| SORLIP1AT | S000482 | GCCAC | CA03g34530, CA11g00460 | phyA, phytochrome, light |
| SORLIP2AT | S000483 | GGGCC | CA06g16770, CA11g08610, CA02g01930 | phyA, phytochrome, light |
| SORLREP3AT | S000488 | TGTATATAT | CA03g34530, CA02g01930 | phyA, phytochrome, light |
| SREATMSD | S000470 | TTATCC | CA06g01320, CA12g19830 | sugar-repressive element (SRE) |
| TGACGTVMAMY | S000377 | TGACGT | CA06g01320, CA06g16770, CA12g19830 | Cotyledons, germinated seeds, seed |
| WRKY71OS | S000447 | TGAC | CA01g18900, CA03g04310, CA03g05890, CA03g05910, CA03g34530, CA03g34540,  CA03g34660, CA03g35880,  CA04g00340, CA04g17190,  CA06g01310, CA06g01320,  CA06g10630, CA06g13860,  CA06g16770, CA06g16790,  CA06g27740, CA07g04080,  CA07g13090, CA09g06930,  CA11g00460, CA11g08610,  CA12g19830 | Gibberellin signaling pathway repressor, defence response |
| XYLAT | S000510 | ACAAAGAA | CA04g17190, CA06g01310, CA02g01930 | Secondary xylem, wood formation |

| **Gene ID** | **NAME** | **Root** | **Stem** | **Leaf** | **Bud** | **Flower** | **1cm_F** | **3cm_F** | **4cm_F** | **5cm_F** | **MG_F** | **Breaker_F** | **B+3_F** | **B+5_F** | **B+7_F** |
| --- | --- | --- | --- | --- | --- | --- | --- | --- | --- | --- | --- | --- | --- | --- | --- |
| CA01g18900 | IAA1 | 0.232176 | 2.577632 | 5.980833 | 0 | 1.152928 | 0.47825 | 1.430638 | 0.705516 | 1.769444 | 0 | 0.212417 | 0.426575 | 0.435282 | 0 |
| CA04g00340 | IAA10 | 38.42817 | 20.44562 | 6.462785 | 11.9765 | 8.779156 | 26.17478 | 25.97369 | 39.79453 | 22.58985 | 6.433725 | 2.246501 | 4.511418 | 7.480704 | 8.091457 |
| CA04g17190 | IAA11 | 277.4706 | 269.1603 | 96.75433 | 73.62367 | 101.6154 | 247.7249 | 201.4629 | 143.8026 | 199.4273 | 149.6336 | 126.6499 | 36.12353 | 37.69042 | 52.7306 |
| CA06g01310 | IAA12 | 6.674167 | 8.92077 | 4.646647 | 4.621234 | 5.553567 | 7.988609 | 11.6707 | 9.135524 | 5.695275 | 4.376216 | 3.630691 | 2.154202 | 1.859995 | 3.962739 |
| CA06g01320 | IAA13 | 0.723121 | 9.38353 | 19.90022 | 3.867993 | 18.46717 | 30.64169 | 10.29072 | 4.290074 | 30.14179 | 0.902233 | 0 | 0 | 0.290508 | 0.226941 |
| CA06g10630 | IAA14 | 32.96087 | 0.110337 | 0.244883 | 0.700187 | 0.162861 | 0.619272 | 0.112272 | 0.221467 | 0 | 0.31827 | 0.100019 | 0.100429 | 0.358677 | 0.300208 |
| CA06g13860 | IAA15 | 1.172489 | 0.591684 | 0.656591 | 14.24881 | 2.814106 | 64.20302 | 67.23003 | 66.70455 | 81.48498 | 74.52696 | 62.21683 | 3.680095 | 0.183181 | 0.214648 |
| CA06g16770 | IAA16 | 5.550243 | 18.20565 | 16.93928 | 54.96261 | 10.05981 | 30.15378 | 54.29227 | 216.5822 | 141.2489 | 5.924723 | 0.126947 | 0.254935 | 0 | 0.609652 |
| CA06g16790 | IAA17 | 64.21735 | 185.3262 | 113.2054 | 81.86755 | 182.9001 | 469.4989 | 332.9323 | 232.6093 | 222.8053 | 57.7409 | 40.44217 | 46.79818 | 28.36152 | 34.49177 |
| CA06g27740 | IAA18 | 2.344978 | 5.325154 | 7.058358 | 7.220678 | 1.601129 | 34.26509 | 25.58755 | 6.086543 | 12.12701 | 0.568908 | 3.620376 | 0.403913 | 0.824316 | 0.321973 |
| CA07g04080 | IAA19 | 2.714094 | 2.410563 | 84.26257 | 1.711568 | 2.695504 | 55.12381 | 21.85255 | 10.8865 | 23.04841 | 33.1863 | 25.32773 | 2.593021 | 14.75627 | 12.8789 |
| CA02g01930 | IAA2 | 0.211259 | 0 | 0 | 0 | 0 | 0 | 0 | 0 | 0.345007 | 0.102506 | 0.09664 | 0 | 0.297051 | 0.116026 |
| CA07g13090 | IAA20 | 0 | 0 | 0 | 0 | 0 | 0 | 0 | 0 | 0 | 0 | 0 | 0 | 0.233849 | 0 |
| CA09g06930 | IAA21 | 16.51612 | 25.08267 | 13.43722 | 5.987645 | 14.15918 | 10.27045 | 16.96169 | 13.7306 | 14.16479 | 8.996686 | 10.26375 | 12.16659 | 13.21827 | 14.71874 |
| CA11g00460 | IAA22 | 0 | 0.278439 | 0.308984 | 0 | 0.27399 | 0 | 0 | 0 | 0 | 0 | 0 | 0 | 0 | 0 |
| CA11g08610 | IAA23 | 4.931028 | 6.856895 | 11.90456 | 1.835537 | 4.788424 | 19.863 | 18.00553 | 17.09282 | 15.62851 | 12.3352 | 15.73968 | 9.160392 | 37.49226 | 24.91526 |
| CA12g19830 | IAA24 | 2.917714 | 3.817314 | 0.121031 | 0 | 0 | 0.890382 | 0.665873 | 0.218916 | 0.470609 | 0 | 0 | 0 | 0 | 0 |
| CA00g43090 | IAA25 | 0.710599 | 13.74619 | 20.55993 | 23.68966 | 10.46836 | 1.463734 | 4.25699 | 2.51919 | 0.902595 | 2.52848 | 0.433416 | 0.217596 | 0.666114 | 0.39027 |
| CA00g82880 | IAA26 | 28.93642 | 46.87956 | 21.88638 | 4.295378 | 11.49529 | 90.18148 | 71.78408 | 28.47238 | 49.99731 | 17.43193 | 13.75262 | 7.940168 | 4.015899 | 2.559269 |
| CA00g93260 | IAA27 | 70.44129 | 198.2489 | 163.1437 | 149.0575 | 166.0379 | 285.9361 | 246.2071 | 97.61755 | 183.8199 | 10.70886 | 3.533613 | 1.182699 | 0.258609 | 0.505055 |
| CA03g04310 | IAA3 | 49.08484 | 66.93024 | 10.26071 | 2.567352 | 2.671157 | 57.82535 | 38.58363 | 16.34571 | 33.85771 | 15.66026 | 20.45443 | 1.235385 | 1.733329 | 2.585013 |
| CA03g05890 | IAA4 | 46.09094 | 118.8468 | 59.65926 | 2.191102 | 17.26609 | 27.27466 | 41.41758 | 1.22857 | 24.76023 | 0.196175 | 0 | 0 | 0 | 0.555125 |
| CA03g05910 | IAA5 | 77.88676 | 83.07722 | 47.03339 | 9.902644 | 16.15982 | 20.94782 | 9.583812 | 7.877059 | 15.24017 | 2.322074 | 1.313515 | 1.099083 | 4.934681 | 3.154017 |
| CA03g34530 | IAA6 | 2.803778 | 0.385881 | 1.141898 | 116.7866 | 65.43745 | 479.8819 | 329.3006 | 158.5212 | 54.94617 | 17.19092 | 2.798358 | 0.117076 | 0 | 0.419964 |
| CA03g34540 | IAA7 | 19.29119 | 62.60772 | 22.89984 | 113.2544 | 200.5391 | 264.7159 | 485.0883 | 690.2226 | 583.681 | 386.9696 | 421.4723 | 1297.297 | 804.5541 | 1616.397 |
| CA03g34660 | IAA8 | 63.90371 | 148.9432 | 22.27598 | 7.500432 | 37.43335 | 320.6094 | 307.0821 | 222.725 | 275.0883 | 26.62847 | 10.55855 | 12.23722 | 6.732634 | 13.82291 |
| CA03g35880 | IAA9 | 257.3854 | 309.0534 | 96.86973 | 19.54529 | 137.9005 | 58.64209 | 41.73183 | 4.717935 | 23.78188 | 23.69151 | 12.26997 | 3.983798 | 3.91456 | 2.11708 |

|  | **CM334 - *Phytophthora capsisi* inoculated** | | | | **CM334 - Virus inoculated** | | | | | | | | | |
| --- | --- | --- | --- | --- | --- | --- | --- | --- | --- | --- | --- | --- | --- | --- |
|  | ***P.capsici*** | | | | **PepMoV inoculated** | | | | **TMV-P0 inoculated** | | | **TMV-P2 inoculated** | | |
| **Gene ID** | **0h** | **1dpi** | **2dpi** | **3dpi** | **Viruses_0h** | **PepMoV_1dpi** | **PepMoV_2dpi** | **PepMoV_3dpi** | **TMV_P0_1dpi** | **TMV_P0_2dpi** | **TMV_P0_3dpi** | **TMV_P2_1dpi** | **TMV_P2_2dpi** | **TMV_P2_3dpi** |
| CA01g18900 | 4.193205 | 0.553025 | 4.203545 | 5.116004 | 2.189992 | 1.653734 | 1.202077 | 2.39845 | 2.679477 | 2.645831 | 0 | 0 | 0 | 0 |
| CA02g01930 | 0 | 0 | 0 | 0 | 0 | 0 | 0 | 0 | 0 | 0 | 0 | 0 | 0 | 0 |
| CA03g04310 | 2.915757 | 0.897278 | 2.77071 | 3.228036 | 4.386372 | 4.486065 | 4.77238 | 3.902986 | 4.895597 | 2.745706 | 1.12693 | 0 | 5.074536 | 10.55399 |
| CA03g05890 | 118.5838 | 32.61964 | 63.47142 | 89.33452 | 103.7016 | 87.29434 | 66.7202 | 71.45246 | 107.8944 | 65.1713 | 73.54944 | 134.2309 | 102.5555 | 93.40175 |
| CA03g05910 | 35.18991 | 41.60669 | 38.71926 | 41.59508 | 39.29514 | 27.32554 | 21.44629 | 26.42665 | 45.58944 | 42.39756 | 54.17444 | 50.58801 | 25.99363 | 24.0138 |
| CA03g34530 | 2.959335 | 0.607126 | 0.576845 | 1.248107 | 1.481316 | 1.234442 | 1.094442 | 0.772486 | 1.650129 | 0 | 0 | 0 | 4.272668 | 0 |
| CA03g34540 | 9.87787 | 60.70017 | 56.31892 | 59.75632 | 2.80064 | 2.423477 | 4.381922 | 6.192798 | 1.549101 | 4.85158 | 9.001334 | 7.580098 | 5.762371 | 4.031733 |
| CA03g34660 | 9.486369 | 4.865467 | 11.83437 | 9.802208 | 11.69004 | 10.28702 | 9.520538 | 7.883692 | 9.422041 | 13.36174 | 7.519572 | 9.314904 | 15.64295 | 8.981771 |
| CA03g35880 | 5.057439 | 40.81087 | 30.56029 | 25.06261 | 11.68095 | 10.88154 | 13.92135 | 16.36739 | 17.18002 | 32.21573 | 45.32578 | 16.81785 | 9.987512 | 9.834177 |
| CA04g00340 | 0 | 0 | 0 | 0 | 0 | 0 | 0 | 0 | 0 | 0 | 0 | 0 | 0 | 0 |
| CA04g17190 | 89.65749 | 134.2712 | 138.2179 | 125.8607 | 115.62 | 110.3265 | 99.53916 | 105.073 | 111.4626 | 99.70427 | 121.607 | 114.4293 | 114.1743 | 124.6913 |
| CA06g01310 | 0.334265 | 0.617188 | 0.293203 | 0.634397 | 0.702861 | 0.188014 | 0.218219 | 0.914666 | 0 | 0 | 0 | 0 | 0 | 0 |
| CA06g01320 | 1.241066 | 0.859316 | 3.537983 | 1.177701 | 2.339383 | 1.69707 | 1.13478 | 1.32935 | 2.944876 | 0 | 4.952863 | 0 | 0 | 2.301704 |
| CA06g10630 | 34.84406 | 23.10256 | 32.23084 | 34.86859 | 20.01939 | 16.64666 | 15.52077 | 18.03288 | 27.868 | 19.33184 | 13.91274 | 18.98802 | 21.00595 | 15.80534 |
| CA06g13860 | 2.881045 | 0 | 0.361019 | 0.390564 | 1.138115 | 1.635654 | 1.579944 | 2.691301 | 4.146271 | 0 | 7.340535 | 3.926514 | 0 | 3.000353 |
| CA06g16770 | 4.172549 | 0 | 1.829991 | 0 | 2.111988 | 4.486055 | 1.929743 | 4.218016 | 4.969872 | 0 | 2.419014 | 3.915955 | 0 | 0 |
| CA06g16790 | 0 | 0 | 0 | 0 | 0 | 0 | 0 | 0 | 0 | 0 | 0 | 0 | 0 | 0 |
| CA06g27740 | 8.267348 | 1.040787 | 3.955509 | 6.062235 | 7.238982 | 4.590318 | 4.80569 | 2.718339 | 3.580777 | 0 | 4.574269 | 11.77092 | 14.10676 | 7.695974 |
| CA07g04080 | 38.09382 | 92.206 | 108.9474 | 89.91655 | 47.57033 | 27.65821 | 34.68206 | 32.59438 | 46.76611 | 87.68416 | 85.02528 | 57.38352 | 62.54494 | 60.66678 |
| CA07g13090 | 0.737829 | 1.702913 | 0.970788 | 1.050237 | 0.496694 | 0.207503 | 0.374067 | 0.428362 | 1.86511 | 0 | 0 | 0 | 4.261263 | 0 |
| CA09g06930 | 7.60803 | 11.03733 | 11.1224 | 9.96991 | 6.701376 | 9.913422 | 7.284982 | 7.050876 | 17.62881 | 11.64799 | 12.92286 | 2.720065 | 10.14453 | 10.10709 |
| CA11g00460 | 0.308683 | 0 | 0 | 0.292923 | 0.473192 | 0.678554 | 0.586493 | 1.522372 | 0 | 0 | 4.07374 | 0 | 0 | 4.031733 |
| CA11g08610 | 9.895179 | 4.176116 | 7.191695 | 12.07281 | 4.676251 | 3.748958 | 7.562498 | 5.77183 | 12.00881 | 11.96762 | 3.441389 | 2.697185 | 5.904484 | 2.06099 |
| CA12g19830 | 0 | 0 | 0 | 0 | 0.720612 | 0.587281 | 0.875378 | 0.958611 | 1.127613 | 0 | 0 | 1.892712 | 2.911863 | 1.870134 |
| CA00g43090 | 27.19532 | 5.359877 | 20.10218 | 15.94804 | 13.25097 | 16.24185 | 14.4444 | 15.22456 | 8.890191 | 4.740882 | 19.55113 | 23.69415 | 30.37907 | 14.27915 |
| CA00g82880 | 6.12678 | 2.828129 | 4.030614 | 5.087222 | 11.73416 | 9.37968 | 8.859034 | 8.994609 | 6.443611 | 7.108742 | 7.660405 | 13.05631 | 12.16334 | 11.92942 |
| CA00g93260 | 9.680313 | 3.723704 | 7.783563 | 7.399889 | 16.51538 | 10.5997 | 12.25992 | 10.3514 | 10.53226 | 9.20003 | 4.016945 | 14.39567 | 9.109913 | 12.93945 |


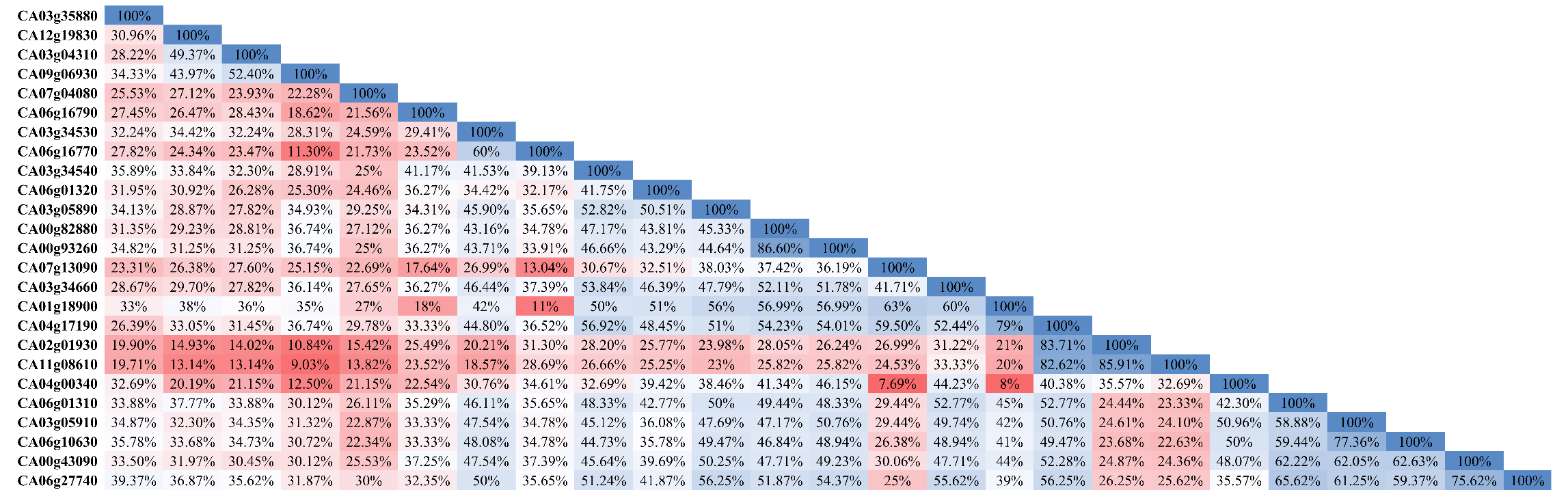


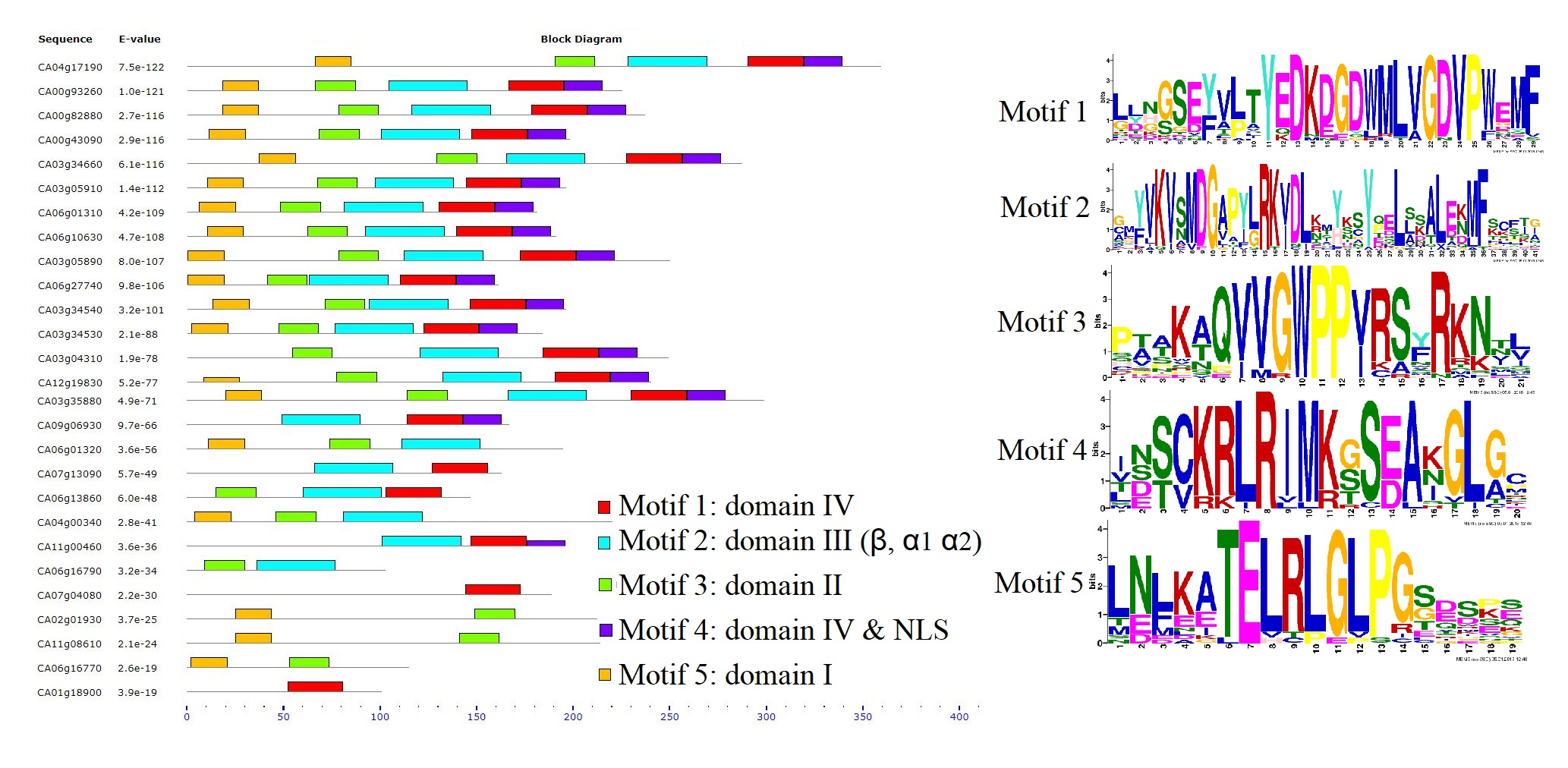


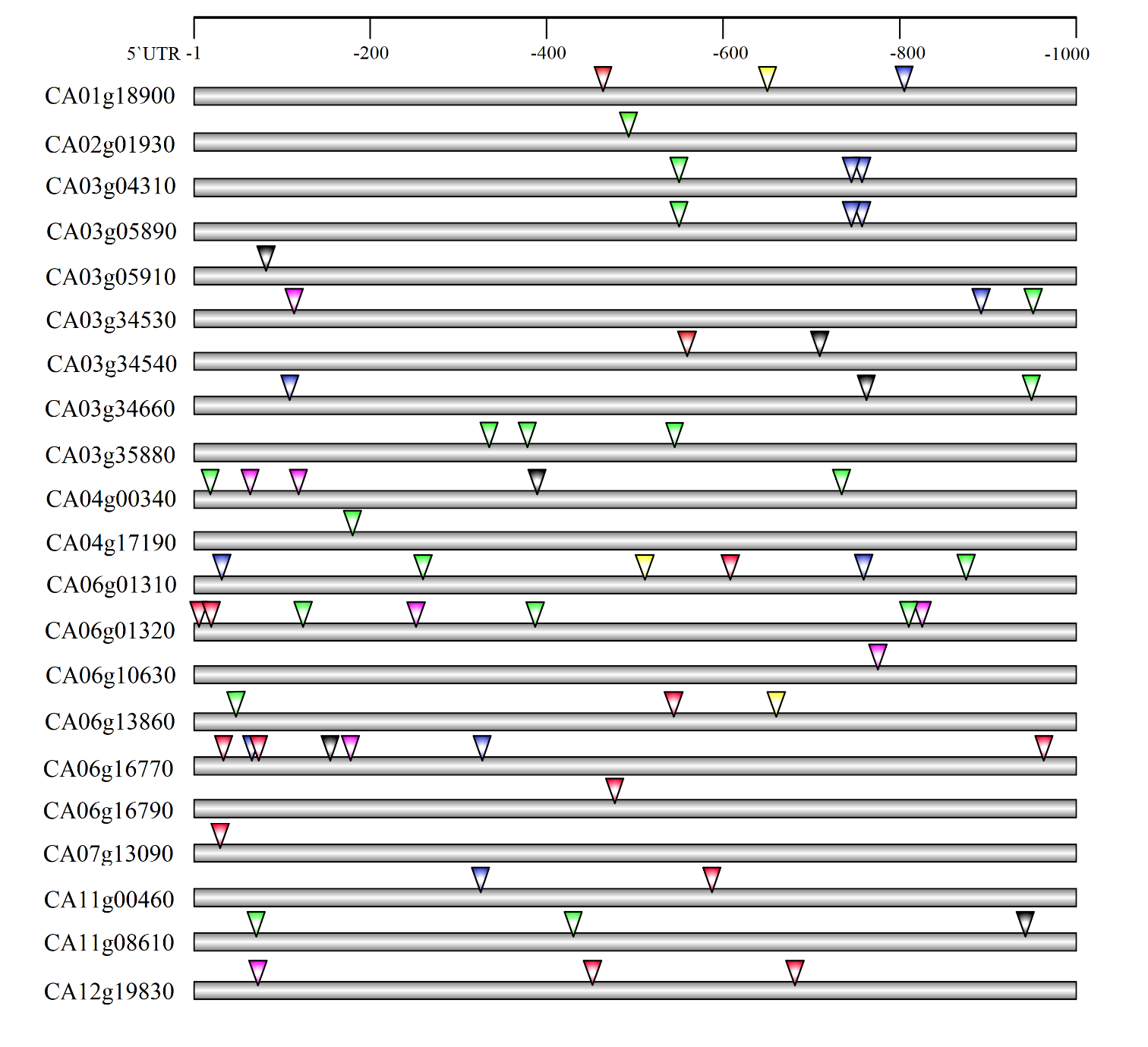


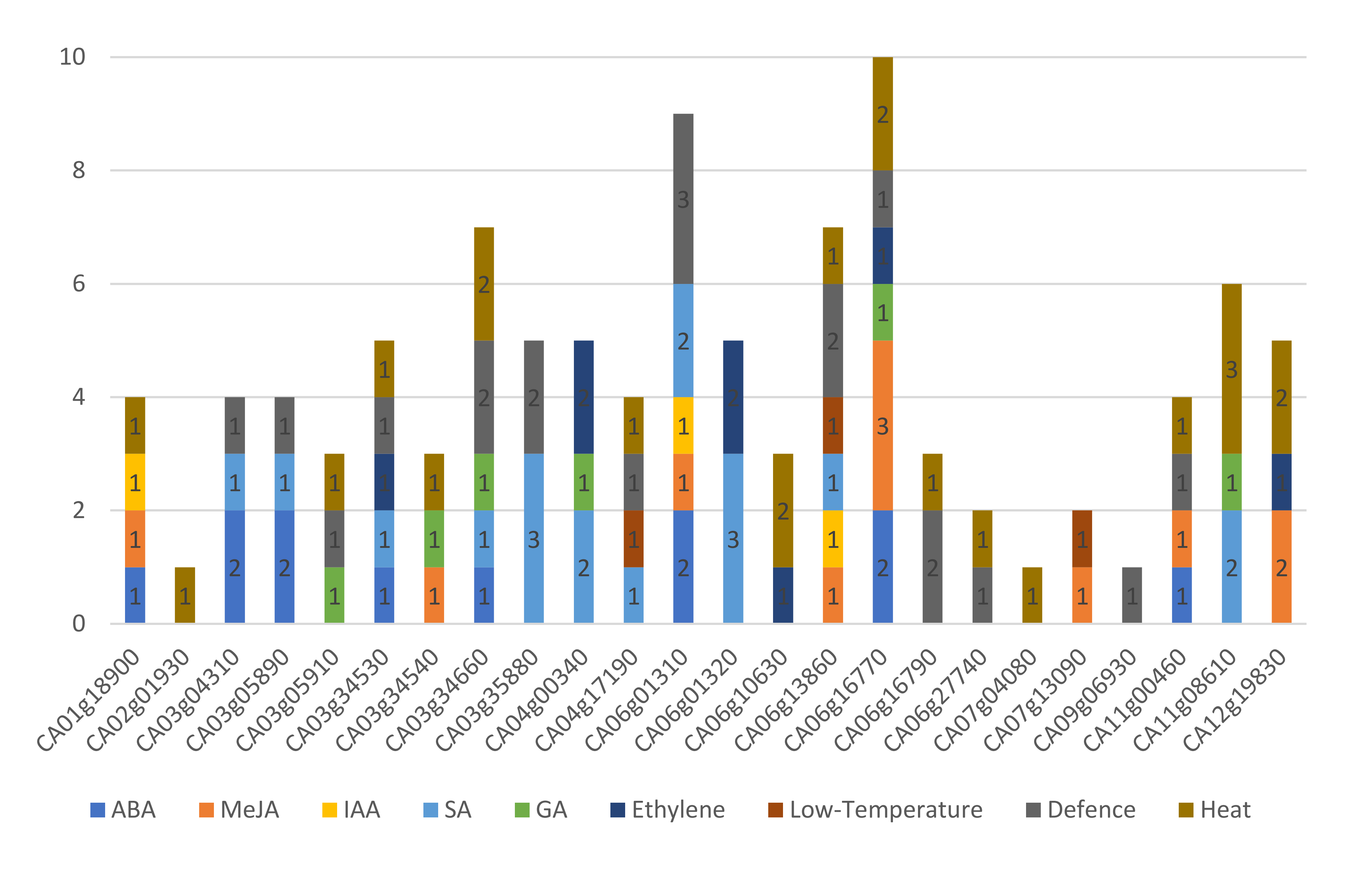


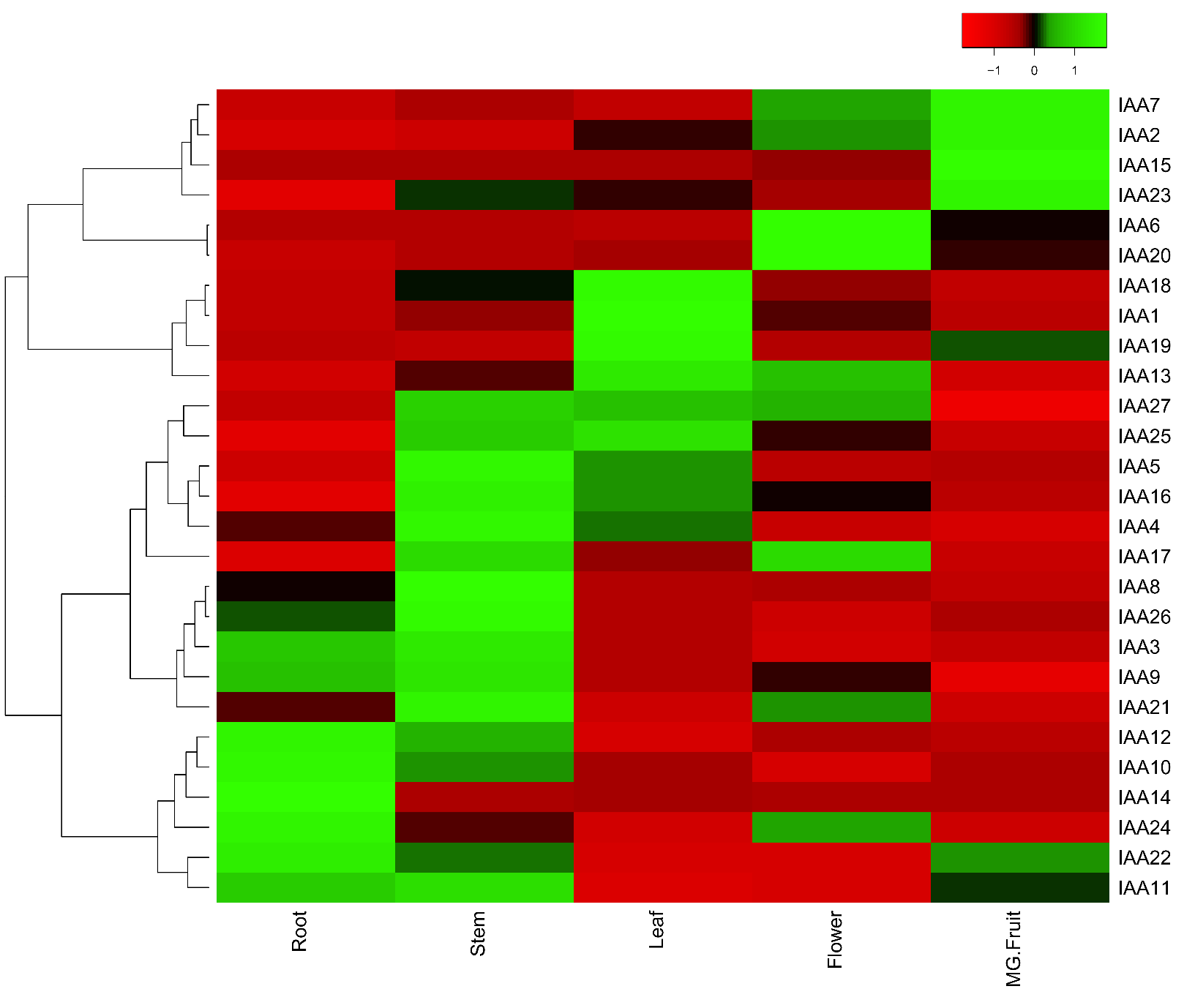


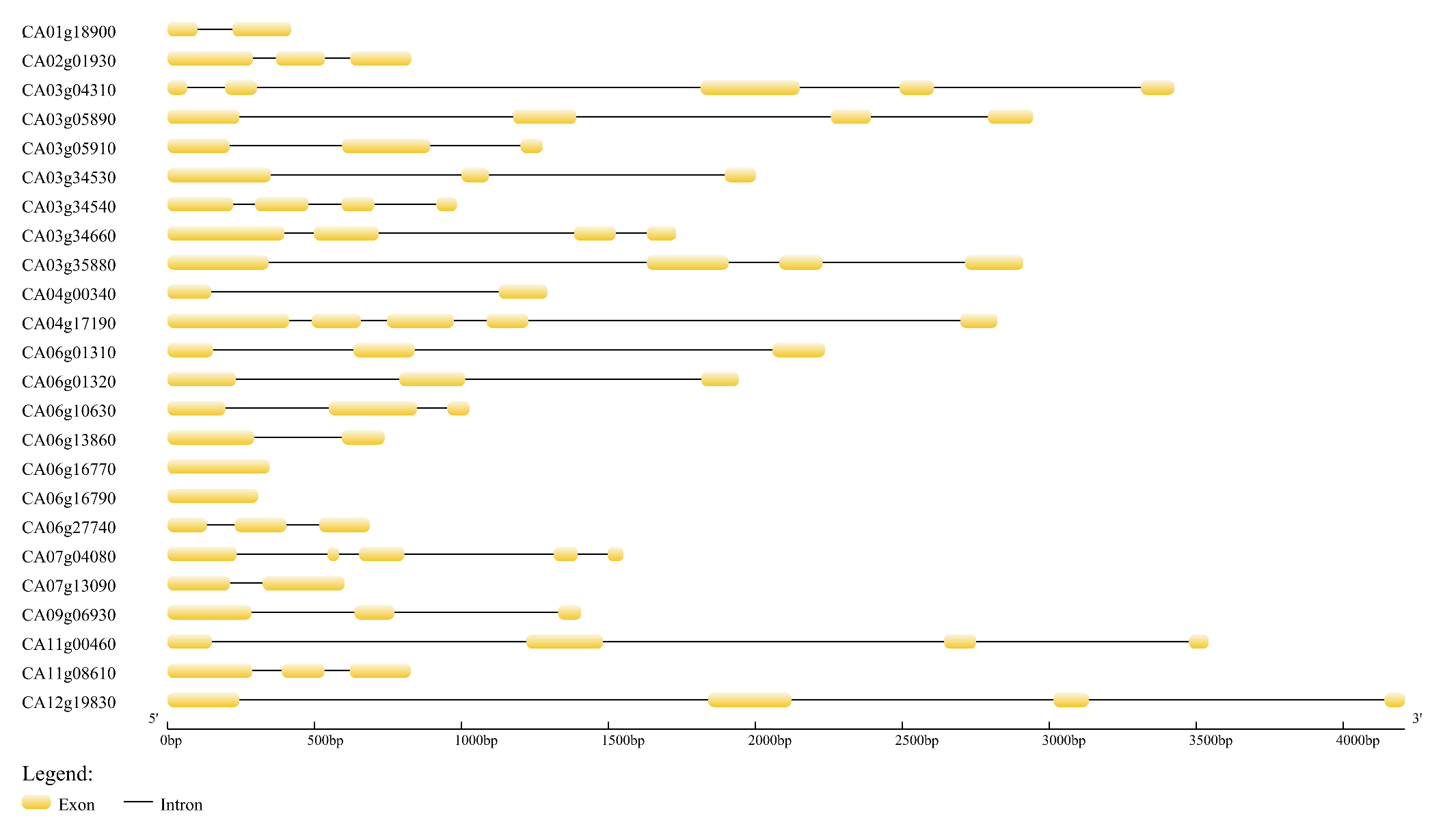

Supplement: Supplementary file 1 — Supplementary information [file 41598_2018_30468_MOESM1_ESM.docx]
